# ﻿Nomenclature and typification of *Cathayaargyrophylla* (Pinaceae)

**DOI:** 10.3897/phytokeys.215.96362

**Published:** 2022-12-15

**Authors:** Chao Tan, David E. Boufford, Yong Yang

**Affiliations:** 1 Co-Innovation Center for Sustainable Forestry in Southern China, Key Laboratory of State Forestry and Grassland Administration on Subtropical Forest Biodiversity Conservation, College of Biology and Environment, Nanjing Forestry University, 159 Longpan Road, Nanjing 210037, China Nanjing Forestry University Nanjing China; 2 Harvard University Herbaria, 22 Divinity Avenue, Cambridge, MA 02138, USA Harvard University Herbaria Cambridge United States of America

**Keywords:** *Cathayaargyrophylla*, gathering, nomenclature, Pinaceae, Shenzhen Code, typification

## Abstract

In 1958, Chun and Kuang described *Cathaya* Chun & Kuang as a new genus of Pinaceae. They included one fossil species, *C.loehri* (Engelh. & Kink.) Chun & Kuang and two extant species, *C.argyrophylla* Chun & Kuang and *C.nanchuanensis* Chun & Kuang. Under Art. 40.1 of the Shenzhen Code, they did not validly publish *Cathaya* because they did not designate a type species for the generic name. Four years later (Chun and Kuang 1962), they again published on *Cathaya* (in Chinese) where they cited the 1958 publication and included one species *C.argyrophylla* (with *C.nanchuanensis* in synonymy) under *Cathaya*. According to Art. 40.3, they validated both the generic name *Cathaya* and *C.argyrophylla* in 1962. Further examination of the type collection and botanical history of the discovery of *C.argyrophylla* revealed that the type collection *Guang-Fu-Lin-Qu Exped. 00198* consists of 11 duplicates in the South China Institute of Botany (IBSC) and 9 duplicates in other herbaria (GAC, IBK, PE, SZ) and that the intended type specimen (IBSC0000004) consists of two gatherings: the bark, reproductive shoot and seed cones collected in 1955, whereas pollen-bearing cones were collected in 1956. We thus lectotypify the name *C.argyrophylla* with the specimen *Guang-Fu-Lin-Qu Exped. 00198* (IBSC0000004 excl. pollen-bearing cones).

*Cathayaargyrophylla* Chun & Kuang is the sole extant species of *Cathaya* Chun & Kuang (Pinaceae). The genus was widely distributed during the Tertiary (Wang et al. 1998; Liu and Basinger 2000), but is presently relictual and locally restricted in Guangxi, Chongqing, Hunan and Guizhou provinces, China, perhaps due in part to unfavorable conditions brought about by the Quaternary glaciations (Liu and Basinger 2000; Manchester et al. 2009). *Cathayaargyrophylla* is represented in nature by fewer than 5,000 living individuals and is considered to be Vulnerable (VU, Qin et al. 2017; Yang 2021). It is listed under the first class of protection in the recently released National Key Protected Wild Plant Species (http://www.forestry.gov.cn/main/3954/20210908/163949170374051.html, accessed 26 August 2022; Yang et al. 2021).

In 1955, J. X. Zhong led a group of botanists to collect an unusual conifer in Guang-Fu-Lin-Qu in Longsheng Xian, Guangxi Province, China (Fu and Cheng 1981, Fu 1998). Specimens with seed-bearing cones were collected on 16 May 1955; pollen-bearing cones were collected in the following year, 1956 (Fu and Cheng 1981; Fu 1998). Chun and Kuang (1958) determined that the collections represented both an undescribed genus and an undescribed species. They examined herbarium specimens deposited in PE and found that similar specimens had been collected in Nanchuan Co., Chongqing, China (Fu and Cheng 1981; Fu 1998). In reporting their discovery, Chun and Kuang (1958) described the new genus *Cathaya* Chun & Kuang with two extant new species, *C.argyrophylla* and *C.nanchuanensis*, and made one new combination to include a fossil species. Under Article 40.1 of the Shenzhen Code, the generic name *Cathaya*, however, was not validly published because they included three species within the genus, but did not designate a type for the generic name (Doweld 2016, 2018). The two specific names were not validly published because of the invalid status of the generic name. When Chun and Kuang (1962) introduced the species to Chinese audiences, they fully cited the Latin description from their earlier paper (Chun and Kuang 1958) for both the generic and specific names and cited only one species, *C.argyrophylla*, under the genus name *Cathaya*. Under Art. 40.3, they validly published *Cathaya* and *C.argyrophylla* at the same time. However, one year earlier, in 1961, the Russian botanist, Karavaev, published *Cathaya* Karav. as the generic name of a fossil plant (Karavaev 1961; Doweld 2016, 2018). When this was noticed, Doweld (2016) proposed conserving the name of the living *Cathaya* of Chun and Kuang against the fossil homonym *Cathaya* Karav. The proposal was approved, and *Cathaya* Chun & Kuang is now a conserved name (appendix III, Turland et al. 2018).

A further complication, however, was recently discovered. Chun and Kuang (1958, 1962) designated Guang-Fu-Lin-Qu Exped. (as ‘*Expeditionis Kwangfu Lingchu*’) *00198* (IBSC) as the type of *C.argyrophylla*. We examined the collection and found 11 duplicates of ‘*Expeditionis Kwangfu Lingchu*’) *00198* in IBSC. Under Art. 40.2 Note 1, these duplicates should be considered as syntypes (duplicates in other herbaria are considered isosyntypes). Of the 11 duplicates, IBSC0000004 (Fig. 1) bears the annotation ‘TYPUS’ and was published as Plate VIII in the paper of 1958. We consider that it was Chun and Kuang’s (1958) intention to indicate this specimen as the type. In examining the specimen further, we found that it includes reproductive shoots of seed-bearing cones, seeds in bags, bark, pollen-bearing cones in a bag and a branch bearing a pollen cone. Prof. L.K. Fu, a retired gymnosperm specialist in China, commented that the seed-bearing cones were collected in May, 1955, while the pollen-bearing cones were collected in 1956 (Fu and Cheng 1981; Fu 1998). Based on these clues, we consider that IBSC0000004 consists of two gatherings, pollen-bearing cones collected in 1956 and other parts of the plant collected in 1955, but which were later mounted on a single herbarium sheet. Here we lectotypify the name *C.argyrophylla* Chun & Kuang with IBSC0000004, excluding the branch with pollen-bearing cones and the bag containing two pollen-bearing cones.

**Figure 1.**
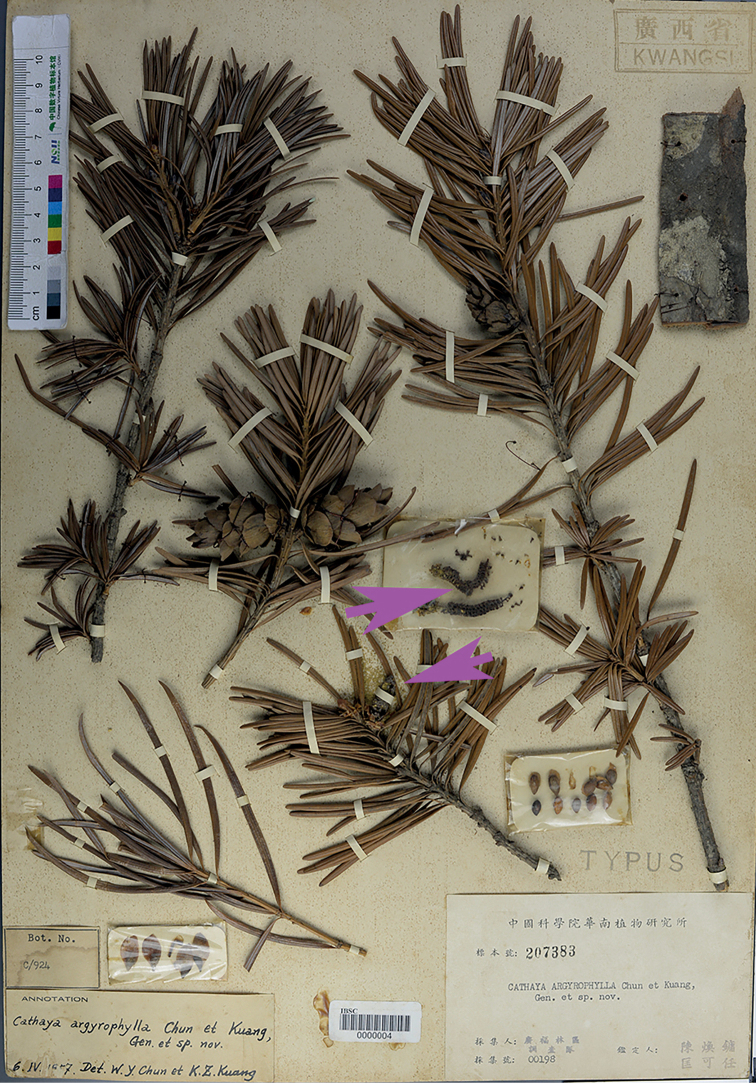
Lectotype of *Cathayaargyrophylla* Chun & Kuang: *Guang-Fu-Lin-Qu Exped. 00198* (IBSC0000004 excluding the pollen-bearing cones in the bag and the adjacent lower left branch with a pollen-bearing cone).

## ﻿Typification

### ﻿ Cathaya

Taxon classificationPlantaePinalesPinaceae

Chun & Kuang, Acta Bot. Sin. 10: 245. Sep 1962 [Pin. / Pin.] nom. cons.

EFBB10FD-0542-5FCF-A0C0-530EC909EB18

#### Type species.

*C.argyrophylla* Chun & Kuang.

### ﻿ Cathaya argyrophylla

Taxon classificationPlantaePinalesPinaceae

Chun & Kuang, Acta Bot. Sin. 10: 245. Sep 1962.

12EEFA09-7596-5531-A7D8-33E9F595517B

#### Type.

China. Guangxi, Longsheng Xian., alt. 1400 m, on sunny rocky slopes, 16 May 1955, *Guang-Fu-Lin-Qu 00198* (lectotype: IBSC0000004 excl. pollen-bearing cones, photo!, here designated; isolectotypes: GAC0005145 photo!, IBK00200027 photo!, IBK00190034 photo!, IBK00190035 photo!, IBK00190036 photo!, IBSC0000005, IBSC0011250, IBSC0003243 photo!, IBSC0003246 photo!, IBSC0003247, IBSC0003248 photo!, IBSC0003249 photo!, IBSC0003250 photo!, IBSC0003251 photo!, IBSC0003252 photo!, PE00000497 photo!, PE00000498 photo!, SYS00095331 photo!, SZ00004502 photo!).

#### Note.

Photos are in Lin (2014, citing PE00000497) and in the Chinese Virtual Herbarium (2022).

## Supplementary Material

XML Treatment for Cathaya

XML Treatment for Cathaya argyrophylla
